# British Gynecological Society

**Published:** 1887-12

**Authors:** 


### Society Reports.

### *BRITISH GYNECOLOGICAL SOCIETY.*

*Meeting of October 12, 1887.*

Dr. G. Granville Bantock, President, in the Chair.

A dermoid cyst of the ovary was presented by Dr. Robert Barnes. He had removed it a month previously from a lady about thirty years of age. She had suffered intense pain in the pelvis for several years, disabling her from active life, and exhausting her strength. There was a tumor in Douglass pouch, the size of a small orange, movable and firm. The uterus was of normal size. Its position and differentiation from the tumor was determined by the sound. Before submitting to operation, the patient, under other advice, went through a course of massage, which, of course, did no good. Dr. Barnes had known at least one case in which a fatal issue had occurred from injudicious massage in abdominal disease. This lady made a good recovery.

Mr. Lawson Tait said that small dermoid tumors of the ovary generally gave rise to the most intense pain, a fact for which some reasonable explanation was required. It was extremely difficult to diagnose between them and disease of the tube. Perfect accuracy of diagnosis in pelvic disease was not possible in the most skilled and experienced hands.

Dr. Heywood Smith considered the pain to be due to pressure upon the contiguous nerves. A tumor that rose into the abdomen, usually caused but little pain. The amount of pain had to be considered when giving an opinion as to the advisability of operating.

Dr. J. St. Clair Boyd described a case of pseudo-hydramnios, in which the superabundance of fluid did not, as in ordinary cases of hydramnios, take origin in the membrane, but had its source in a hydrocephalic head, from an aperture in which it flowed. The patient, aged forty-two, had had six children, and thought herself pregnant again, but her enormous size seemed inconsistent with that view. She was suffering from enormous abdominal distension, dyspnœa, and dropsical effusion of the lower extremities. On making a vaginal examination, the os uteri was found dilated to the size of a crown piece (silver dollar), and a bag of membrane protruding from it. On rupturing this, thirty pints of fluid escaped. The child was subsequently delivered with the short forceps. The placenta was adherent, and had to be removed. The uterus was then injected with a hot solution of hydrarg. perchlor., 1 to 2000. No hemorrhage occurred. On the third day, all trace of dropsical effusion had disappeared from the patient's lower extremities. The child, which was born at the seventh month, had an immense hydrocephalic head. The fluid, apparently, exuded below the occipital eminence, at which part the skin had become wrinkled, owing to its escape.

Dr. Bedford Fenwick said the interest of the case centered in the fact that a distinct fistulous opening existed; otherwise, the child's head might have been punctured with and through the uterine membranes. Then the case would have been much simpler, and exactly on a par with his own experience.

Dr. Aveling had seen cicatrices on the surface of a hydrocephalocele, which proved that these cysts ruptured in utero, and discharged their contents.

Dr. Cordes, of Geneva, gave notes of a case of monstrosity under his care at La Misericorde Maternity. The cranial vault was represented by a fibrous membrane. On the left side, through an opening as large as a crown piece, the cerebral substance protruded, covered only by the arachnoid and the pia mater. Feet and hands were malformed, and many of the fingers and toes rudimentary. The monstrosity, which was attributed to maternal impressions, lived six days.

Dr. Manselt Moullin showed a specimen of hæmato-salpinx which he had removed from a young married woman aged twenty-five. She had one child two years previously. In the recent state, the tumor was about the size of an egg, constricted in the middle, and filled with blood-clot. The chief symptom had been a constant aching pain in the lower part of the back, and around the pelvis, from which the patient had been unable to obtain relief in any position, sitting or lying. The pain was also much increased on defecation. The attached ovary was, apparently, healthy, and the appendages on the opposite side seemingly so, and were allowed to remain. Numerous adhesions were broken down, but no drainage tube had been required. The patient had made a good convalescence, and was now free from pain.

Dr. Bedford Fenwick said that the menorrhagia was usually a symptom in all inflammatory diseases of the fallopian tubes. It was of great importance to remove the cyst without rupturing it, a matter of some difficulty in the present instance, and he thought the operator was to be congratulated on the successful result of a very unpromising case.

Mr. Lawson Tait was amazed at a recent paper by Mr. Barton, of Liverpool, in which he asserted that such cases as that belonging to the preparation exhibited by Dr. Manselt Moullin ought, under no circumstances, to be touched. Why should a woman, with the symptoms just described, be obliged to go on living the life of an invalid, with a perfectly useless organ inside her—an organ which could never, by any possibility, resume its original functions—any more than a woman, with a cataract in her eye, be prevented from having her sight restored by the removal of a useless lens?

